# Life-history consequences of bidirectional selection for male morph in a male-dimorphic bulb mite

**DOI:** 10.1007/s10493-018-0320-5

**Published:** 2018-11-12

**Authors:** Tom P. G. Van den Beuken, Isabel M. Smallegange

**Affiliations:** Department of Evolutionary and Population Biology, Institute for Biodiversity and Ecosystem Dynamics, University of Amsterdam, PO Box 94240, 1090 GE Amsterdam, The Netherlands

**Keywords:** Major, Minor, Sneaker, ARP, Alternative reproductive phenotype, Population dynamics

## Abstract

Intralocus sexual conflict (IASC) arises when males and females have different trait optima. Some males pursue different alternative reproductive tactics (ARTs) with different trait optima, resulting in different strengths of IASC. Consequently, for instance daughter fitness is differentially affected by her sire’s morph. We tested if—and which—other life-history traits correlatively change in bidirectional, artificial selection experiments for ARTs. We used the male-dimorphic bulb mite *Rhizoglyphus robini*, the males of which are high-fitness ‘fighters’ or low-fitness ‘scramblers’. Twice in each of the five generations of selection, we assessed clutch composition (number of mites of the various life stages present) and size (total number of offspring). Furthermore, we tracked offspring from egg to adulthood in the first and final generation to detect differences between selection lines in the size and duration of stages, and in maturation time. We found that selection for male morph increased the frequency of that morph. Furthermore, compared to fighter lines, scrambler lines produced more females, which laid larger eggs (in the final generations), and maintained a higher egg-laying rate for longer. Otherwise, our results showed no consistent differences between the selection lines in clutch size and composition, life stage size or duration, or maturation time. Though we found few correlated life-history trait changes in response to selection on male morph, the differences in egg laying rate and egg size suggest that IASC between fighters is costlier to females than IASC with scramblers. We hypothesize that these differences in reproductive traits allow fighter-offspring to perform better in small, declining populations but scrambler-offspring to perform better in large, growing populations.

**Electronic supplementary material:**

The online version of this article (10.1007/s10493-018-0320-5) contains supplementary material, which is available to authorized users.

## Introduction

Intralocus sexual conflict (IASC) can arise between males and females when both sexes have different fitness optima for the same phenotypic traits (Bonduriansky and Chenoweth 2009). IASC occurs when males and females (1) share a genetic architecture for a certain phenotypic trait, (2) have different phenotypic trait optima, and (3) neither sex is at its optimal trait expression (Morris et al. 2013). IASCs revolve around sex-specific trait optima for life-history, morphology, behaviour or physiology (Adler and Bonduriansky 2014). As a consequence of the sex-specific trait optima, it is possible that a high-fitness parent produces high-fitness offspring of the same sex, but low-fitness offspring of the opposite sex (Fedorka and Mousseau 2004; Pischedda and Chippindale 2006; Calsbeek and Bonneaud 2008). However, the strength of intersexual conflicts over traits, and their consequences for offspring fitness, may differ within sexes.

In some species there is discrete phenotypic variation *within* sexes; this may generate different levels of IASC between sexes. Large within-sex variation can occur when members of the same sex pursue different life-history strategies. A prominent example of this are male-dimorphic species where two male morphs each pursue different alternative reproductive tactics (ARTs) (Oliveira et al. 2008). Individuals with different ARTs differ in terms of life-history, physiology, morphology and/or behaviour (Gross 1996). In male-dimorphic species, males express one of two ARTs: males are either a ‘major’ or a ‘minor’. Majors typically use precopulatory attributes such as weaponry or ornaments to, respectively, compete with other males or bias female choice (Oliveira et al. 2008). Minors are typically unarmed and rely on various tactics to sire offspring, for instance involving sneaking or sperm competition (Oliveira et al. 2008). ART determination can be fully under genetic control (in alternative strategies) or based on a conditional strategy (Gross 1996). In a conditional strategy an individual estimates the quality of its environment using an environmental cue (such as hormone level, often body size is used as a proxy), there is genetic variation in the sensitivity for this cue. This environmental cue is compared to a genetic threshold of male morph expression to determine whether a male becomes a major or a minor (Gross 1996; Roff 1996; Tomkins and Hazel 2007). At the threshold, the fitness of majors and minors is equal, but majors have the highest mean fitness (Gross 1996). Because of the different ARTs pursued, each of the two morphs has inherently different trait optima (Engqvist and Taborsky 2016). For example: to compete with rivals, majors can benefit from having a large body, whereas minors may sneak matings more easily if they have a small body (e.g., in the horned dung beetle *Onthophagus taurus*; Moczek and Emlen 2000). Given the different optimal trait values of minors and majors, the level of IASC can differ between females and majors, and between females and minors.

Male morphs are known to have opposing effects on their daughters’ fecundity. For example, Harano et al. (2010) found in broad-horned flour beetles (*Gnatocerus cornutus*) that in lines selected for males with large mandibles (a male-limited weapon), female lifetime reproductive success decreased, whereas it increased in lines selected for males with small mandibles. Similarly, after bidirectional selection for male morph expression in the male-dimorphic bulb mite *Rhizoglyphus robini*, female fecundity and longevity decreased in major-lines and increased in minor-lines (Plesnar-Bielak et al. 2014). These results suggest that female fecundity is more negatively affected by the IASC with majors than by the IASC with minors. In a conditional strategy, majors have the highest mean fitness (Gross 1996). Therefore, these results—where the high-fitness majors sire daughters with a lower fecundity—are comparable to other studies where high-fitness fathers sired low-fitness daughters (i.e., the fitness of a parent negatively correlates with the fitness of offspring of the opposite sex). These negative correlations are likely a result of a higher IASC between high-fitness fathers (or mothers) and daughters (or sons) (Fedorka and Mousseau 2004; Pischedda and Chippindale 2006). Though IASC will, to varying degrees, concern many traits, some specific traits have been suggested to explain negative correlations between sire and daughter fitness. These traits include inheritance of major-specific high expression levels of metabolic genes (Plesnar-Bielak et al. 2014; Stuglik et al. 2014), male-associated hormone production (Mills et al. 2012) and possibly the expression of male-specific morphological traits in females (Swierk and Langkilde 2013; Buzatto et al. 2018). However, if female fecundity is affected by IASC, it seems likely that other life-history traits in offspring of high- or low-fitness males are affected as well.

Previous studies have shown that selection for particular traits may lead to correlated responses in other life-history traits (Roff 1993). For example, bidirectional selection for testis length in the fruit fly *Drosophila hydei* led to correspondingly long or short testes in adult males, but also led to longer maturation time of both males and females in lines selected for long testes (Pitnick 2000). Here, we aimed to investigate in detail to what extent bidirectional selection for male morph expression in the male-dimorphic bulb mite *R. robini* affects key correlated life-history traits such as the size and duration of life stages and male weapon size.

The bulb mite *R. robini* is a cosmopolitan species that feeds off the roots and tubers of a large number of plants, several of which are of economic importance including onion, garlic, rye and several ornamental plants (Díaz et al. 2000). *Rhizoglyphus robini* mites go through the following life stages: egg, larva, protonymph, deutonymph (facultative dispersal stage), tritonymph and adult (Baker 1982). As in many mite species, adult bulb mite females are larger than conspecific males (Walter and Proctor 2013), and even though female tritonymphs are on average larger than male tritonymphs, it is not possible to distinguish sexes or morphs before the adult stage with certainty. Adult male bulb mites are dimorphic: males develop as high-fitness ‘fighters’ (majors) which have an enlarged third leg pair that they can use as weaponry to kill rivals; or males develop as low-fitness ‘scramblers’ (minors) which, like females, do not have an enlarged third leg pair (Radwan and Klimas 2001). Scramblers can instead increase their fitness by making a larger investment in postcopulatory attributes (Van den Beuken and Smallegange, in press). Male morph expression in bulb mites is determined by a conditional strategy which is affected by both environmental and genetic factors (Tomkins and Hazel 2007). If juvenile males grow up in favourable food conditions, they are likely to grow larger; if they surpass a genetically determined size-threshold for male morph expression they will develop into fighters, otherwise they will become scramblers (Smallegange et al. 2012).

We conducted a bidirectional selection experiment for male morph expression. The experiment lasted five generations; twice within each generation we assessed female fecundity, measured as clutch size (the total number of offspring), and the number of mites of each stage present in the clutch (clutch composition). Additionally, we isolated eggs and assessed the mean size and duration of each life stage of these individuals, as well as the total time they required to maturate.

Selection for high- or low-fitness parents can impact several offspring life-history traits. Firstly, high-fitness parents can sire high-fitness offspring of the same sex, but low-fitness offspring of the opposite sex (Fedorka and Mousseau 2004; Pischedda and Chippindale 2006; Calsbeek and Bonneaud 2008). Such impacts on offspring fitness can be the result of higher IASC, as the traits that for instance grant a high fitness to fathers, result in a low fitness in daughters (often egg production is used as a measure of female fitness) (Bonduriansky and Chenoweth 2009). Secondly, bidirectional selection for fighter leg size in *R. echinopus* resulted in correlated responses in the leg sizes of scramblers and females; strongly suggesting that the expression of enlarged legs (i.e., a fighter-limited weapon) is not uncoupled between morphs or sexes (Pike et al. 2017; Buzatto et al. 2018). Lastly, other studies selecting bidirectionally for male weaponry (or weapon size) have found that female clutch size decreased in lines selected for weapons, and increased in lines selected against weapons (Harano et al. 2010; Plesnar-Bielak et al. 2014). It is therefore possible that the decrease in female fecundity is a consequence of the expression of male-limited (or fighter-limited) traits in females; such expressions can be costly to female fitness (e.g., Swierk and Langkilde 2013).

Here, we hypothesize that female clutch size would be smaller (or larger) in lines selected for fighter (or scrambler) males (cf. Harano et al. 2010; Plesnar-Bielak et al. 2014). In accordance with the environmental threshold model (Hazel et al. 1990, 2004), we expect that successful selection for male morph will be the result of a shift in the threshold for male morphs, and not a result of a shift in the mean size distribution of the male part of the population (Smallegange 2011). Furthermore, we hypothesize that this decrease in female fecundity is linked to the (limited) expression of fighter-specific weapons in homologous structures of females or scramblers. Therefore, we measured the leg widths of adult females, fighters and scramblers in both selection lines. We obtained these leg width measurements using adults obtained from eggs that we isolated and tracked to adulthood. While tracking these eggs to maturity, we also measured the duration and mean body size of each stage, and the maturation time (i.e., total development time) of individual offspring, and compared them between selection lines. Finally, we also compared the number of mites in each life stage (clutch composition) and compared them between selection lines.

## Materials and methods

### Selection lines

**Fig. 1:**
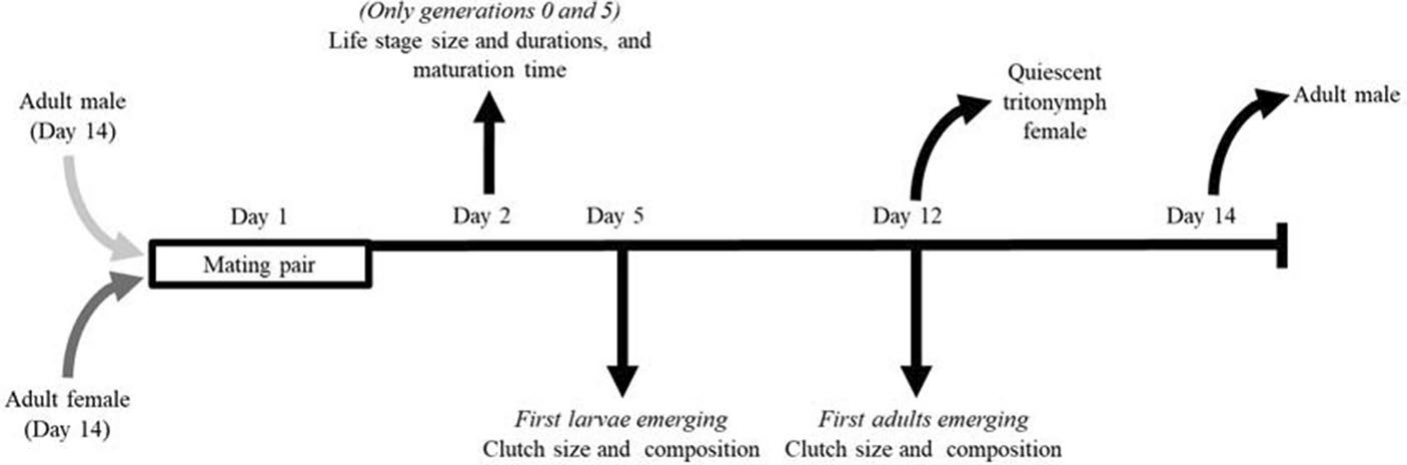
Method used to create fighter and scrambler selection lines and timing of data collection

To found our four stock cultures, four groups of 50 bulb mites each were obtained from flower bulbs in storage rooms near Anna Paulowna in North Holland (The Netherlands) in 2010. These four stock cultures have been intermixed approximately every 6 months to reduce the level of inbreeding. In order to obtain virgin females to start the first generation, we collected 240 quiescent tritonymphs (moulting stage immediately preceding adulthood) from our four stock populations (60 from each) and stored each in 7-mL ‘individual tubes’ (50 × 16 mm) (the maintenance of the stock cultures is described in Van den Beuken and Smallegange 2018). Individual tubes were filled for about two-thirds with plaster of Paris, with charcoal for contrast. The day after the quiescent tritonymphs were collected, the adults emerged. Emerged males were discarded and females remained in their tubes without food. The next day (day 1 in Fig. 1), we selected 60 of these females and used them to start the first generation of our selection experiment, comprising five fighter and five scrambler replicate lines, each consisting of six couples of a male and a female. On the same day (day 1 in Fig. 1), a total of 60 adult males was collected directly from the stock populations—males did not need to be virgin to start a selection line, so we could collect adults rather than quiescent tritonymphs. Each male was transferred to a different 12-mL ‘clutch tube’ (40 × 23 mm, filled for about two-thirds with a mixture of plaster of Paris and charcoal), after which one individually isolated, virgin female (from a different stock culture than the male) was added. Each stock population culture was nearly equally represented in each replicate line. There were some small deviations in the equal distributions as two mites from stock population III were lost (from stock populations I, II, III and IV, we obtained, respectively, 30, 31, 28 and 31 males or females). Water and ad libitum yeast were added to the clutch tubes before adding the male and female. We collectively refer to all offspring produced by the male and female as a ‘clutch’.

To start a new generation, 11 days after the previous generation was started (day 12, Fig. 1), four quiescent tritonymphs were collected from each clutch and stored individually in individual tubes without food. Two days later (day 14, Fig. 1), the females that emerged from these quiescent tritonymphs were selected, and one female from each clutch tube was coupled with one adult male. This adult male was obtained directly from a different clutch, but within the same selection line and replicate, to start a new generation (days 14 and 1, Fig. 1). Scramblers were always picked in scrambler lines, and fighters in fighter lines; aside from their morph, they were randomly selected within each clutch. If none of the collected quiescent tritonymphs on day 12 (Fig. 1) produced an adult female, or if no adult male of the desired morph was present in a population, a replacement was obtained from another clutch within the same replicate and selection line (never from the same clutch). Though inbreeding was unavoidable in our setup, we avoided high levels of inbreeding by making sure that siblings never mated with each other; the coupling of mites with shared grandparents was mostly avoided. In total, we used five generations, each replicated 5 × for both fighter and scrambler selection lines. No control line was established because a random selection of a male morph will result in selection lines very similar to the fighter lines, given the fact that fighter frequencies in the stock populations are very high. Also, alternating morph selection every generation would be ineffective because the control line most likely would not contain scrambler offspring.

For each generation, cleaned or fresh individual and clutch tubes were used. Before the introduction of a mite, the plaster in the clutch tubes was nearly saturated with water, and ad libitum yeast was provided. Within each generation, we added water and ad libitum yeast to each clutch tube on days 5, 9 and 12. If feeding or watering coincided with obtaining data, the feeding and watering was done directly after the observations were made.

### Male morph ratio

To validate that bidirectional selection was effective, we counted the fighters and scramblers in a clutch on day 12; this is when the first adults emerge, and the consequences of male–male killing are minimized. Collected data were not used if one or both parents had died before this assessment was made (*n* = 76), or if no adult males were (yet) present in a clutch (*n* = 32; in total 108 of 300 data points were omitted).

### Maturation time, life stage duration and life stage size

Of the offspring produced by the first and final generation (generations 0 and 5), we isolated three eggs of each clutch. These eggs were collected 2 days after the male and female were paired (day 2, Fig. 1). We recorded (1) the egg length and structural size of each subsequent stage of each mite, (2) the leg width of each adult, and (3) the life stage duration and maturation time. We checked the stage of all collected offspring once a day. If a mite had entered a new stage, it was measured. The structural body size used here was the distance between the ventral postero-distal points of the border between the coxa and trochanter of the anterior leg pair (see *Online Appendix* Fig. A1). Because this region is sclerotized it will not, or only minimally, expand when a mite grows during a stage or starts to produce eggs. Therefore, we feel that this structural body size measurement is the best, non-intrusive, proxy of mite condition. As we cannot take the same measurement for eggs, we have used the length of the elliptical eggs as a measurement. In adults, we measured the width of the basal part of the trochanter of either leg of the third leg pair as an indication of the size of weapons in fighters or expression of homologous structures (see *Online Appendix* Fig. A1). This measurement was taken using the same photographs used to measure structural body size in adults. The structural size measurements and leg widths of adults were also used to see if there were differences in the allometric relation between the leg width and body size between the two selection lines. Photographs for measurements were taken using a Zeiss Stemi 2000-C microscope equipped with a Zeiss Axiocam 105 color camera, 0.63-5 × magnification. We used the Zen 2.3 (Blue edition) software to measure distances on these photographs to the nearest 2.0 µm for third leg width and to the nearest 1.4 µm in structural body size (uncertainty calculated from 10 repeated measurements on a fighter).

Data were omitted if a mite could not be followed from egg to adulthood, as mites that develop into different sexes or morphs can already differ in size during juvenile stages (e.g., Smallegange 2011) (108 omitted data points). Seven additional data points were omitted because the photographs were not clear enough to take accurate measurements. One male developed both a fighter and a scrambler leg and was omitted as well. For the replicate numbers per stage, we refer to *Online Appendix* Table A1.A.

### Clutch size and composition

We measured the clutch size (total number of offspring) and composition (number of individuals of each stage) on day 5 (when the first eggs start hatching) and on day 12 (when the first adults emerge). We excluded the founding generation (generation 0) from our analyses because the founding males and females have been reared in the stock cultures until adulthood (males) or the quiescent stage directly preceding adulthood (females); hence there are various factors that could affect female fecundity (e.g. male age, food availability during development). Clutch size and composition data were omitted if one or both parents had died, or if no offspring was produced before the data were collected (see *Online Appendix* Table A2.B for replicate numbers).

### Statistical analysis

#### Male morph ratio

We estimated the heritability of male morph expression, using data obtained during five generations of selection, with the threshold model of quantitative genetics (Falconer and Mackay 1996). This model calculates the heritability of a normally distributed character (such as hormone level) that is assumed to underlie male morph expression (the environmental cue). In turn, this character is assumed to be based on a threshold of expression such that individuals that are above this threshold express one phenotype whereas those below the threshold express the alternative phenotype (Roff 1996). For this model, we used the proportion of each morph as assessed on day 12 in generations 1 to 5 (for results and formulae, see *Online Appendix* Table A2). To test for differences in male morph ratios between generations 1 and 5 we used a generalized linear mixed-effects model (GLMM, function ‘glmer’ in R’s lme4 package; Bates et al. 2015) with Poisson error distribution, as the data were not normally distributed. In this model we used the number of fighters as a response variable. This response variable was tested against an offset (using R’s ‘offset’ function; R Core Team 2017) of the total number of males as well as generation number (G), selection line (L) and the two-way interaction G × L. The replicate block number was included as a random term (*Online Appendix* Table A3.A).

#### Maturation time, life stage duration and life stage size

We tested how the mean life stage size (egg length, structural size and adult leg width), life stage duration and the total maturation time (from egg to adult) was affected by the selection line (L), generation number (G) and (eventual) morph or sex of tracked offspring (O), the three two-way interactions (G × L, G × O and L × O) and the three-way interaction (G × L × O). The effects of these factors on the life stage size were analysed using a linear mixed-effects model (LMM). The data of the effects of the factors on life stage duration and maturation time were not normally distributed and therefore analysed using a GLMM with Poisson error distribution. In these models the replicate number was included as a random term (see *Online Appendix* Table A3.B and A3.C).

To test if the allometric scaling between the response variables leg width and body size differed between selection lines in females, scramblers or fighters, we used Standardised Major Axis estimation and Testing Routines (SMATR) (Warton et al. 2012).

#### Clutch size and composition

We used a linear mixed-effects model (LMM) to analyse the effects of the generation number (G), selection line (L) and their two-way interaction on the clutch size and the number of mites of each stage per clutch as assessed on days 5 and 12 of each generation (see Fig. 1). The replicate number was included as a random factor (see *Online Appendix* Table A3.D and A3.E).

#### Model simplification procedure

In all analyses using an LMM or a GLMM, we used a model simplification procedure to find the minimal adequate model (see Crawley 2007). During this procedure, we first removed the least significant term of the highest order interaction and compared this reduced model to the full model where this term was not removed. If the removal of the term led to a significant increase in the difference in deviance (likelihood ratio test compared to a χ^2^ distribution, *p* < 0.05) the term was kept in the model and a new reduced model was made. In this new reduced model, the second least significant term of the highest order was removed and compared to a full model. If the removal of a term did not lead to a significant increase in the difference in deviance (*p* > 0.05), then the term was removed from the model and the next least significant term of the highest order was removed from the new reduced model. These steps were repeated until only terms remained in the model of which the removal would lead to a significant increase in the difference in deviance (for the model simplification steps and results see *Online Appendix* Table A3) (Crawley 2007). The random factor ‘replicate block number’ was never removed from a model. We visually inspected Q–Q plots of the residuals of each LMM model to confirm that the data had normally distributed errors. Models with a Poisson error distribution were checked for overdispersion. In the “Results” section, we will report the parameter estimates ($\hat {e}$) of each significant main effect in the best-fitting, minimal model. Parameter estimates of the main effect selection line (L) show the difference in the intercept (Δ) between individuals from scrambler lines compared to individuals from fighter lines (L: Δ scramblers − fighters). Similarly, parameter estimates of the main effect ‘offspring morph or sex’ (O) show the difference in the intercept of scramblers (O: Δ scramblers − females) or fighters compared to females (O: Δ fighters − females). The parameter estimates of the main effect ‘generation’ show the difference in the intercept between generations 5 and 0 (G: Δ generation 5 − 0).

All statistical analyses were performed using the R software v.3.4.3 (R Core Team 2017) integrated in RStudio v.1.1.419 (RStudio Team 2017). The package ‘lme4’ (Bates et al. 2015) was used to run LMMs and GLMMs (using the ‘lmer’ and ‘glmer’ functions, respectively). The package ‘smatr’ (Warton et al. 2012) was used for SMATR. We made figures from our data using the package ‘ggplot2’ (Wickham 2016). The datasets generated and analysed are available in the Figshare repository (doi: 10.6084/m9.figshare.6608663).

## Results

### Male morph ratio

**Fig. 2:**
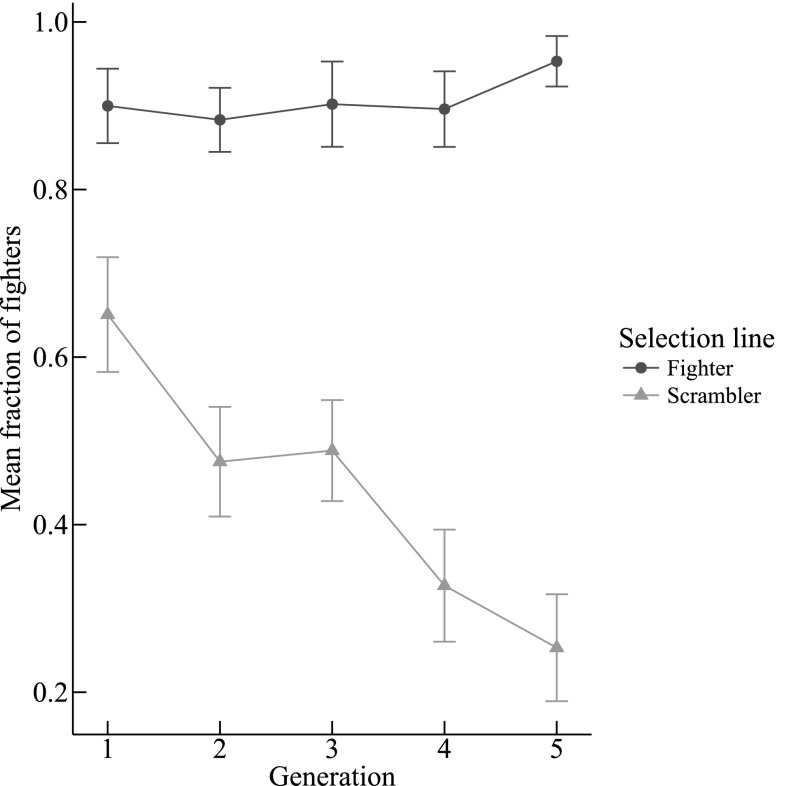
The effect of selection lines on the mean (± SE) fraction of *Rhizoglyphus robini* fighters for each generation of selection

The proportion of males that were fighters increased over the generations in fighter lines and decreased in scrambler lines (G × L: χ^2^ = 284.140, *df* = 1, *P* < 0.001, *n* = 271, Fig. 2). The estimated heritability of the male morph was 0.298 in scrambler lines and 0.419 in fighter lines (see *Online Appendix* Table A2).

### Maturation time, life stage duration and life stage size

**Fig. 3:**
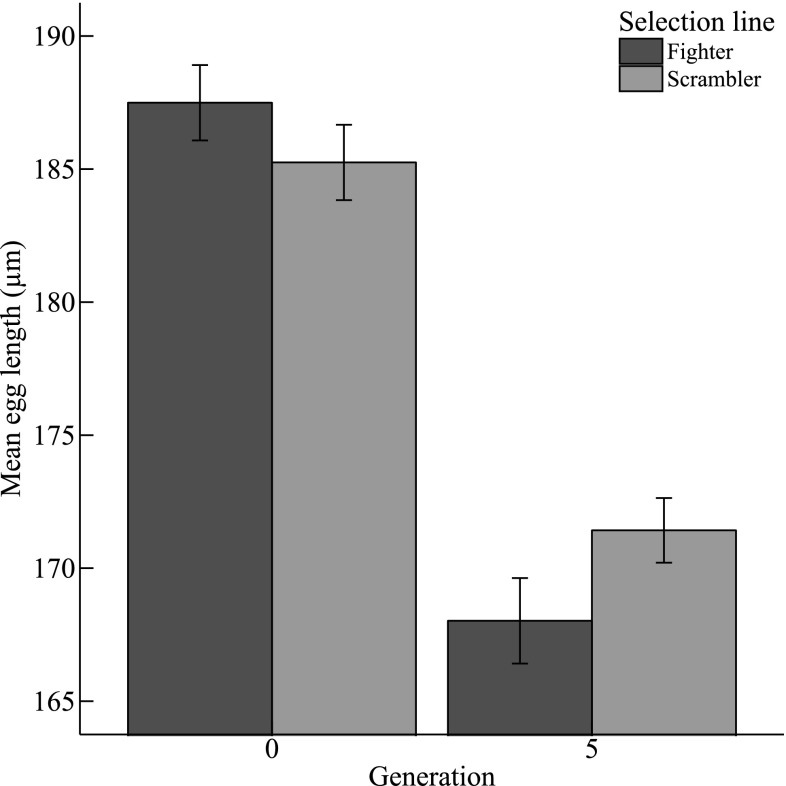
Mean (± SE) *Rhizoglyphus robini* egg length (µm) in clutches obtained from generations 0 and 5

**Fig. 4:**
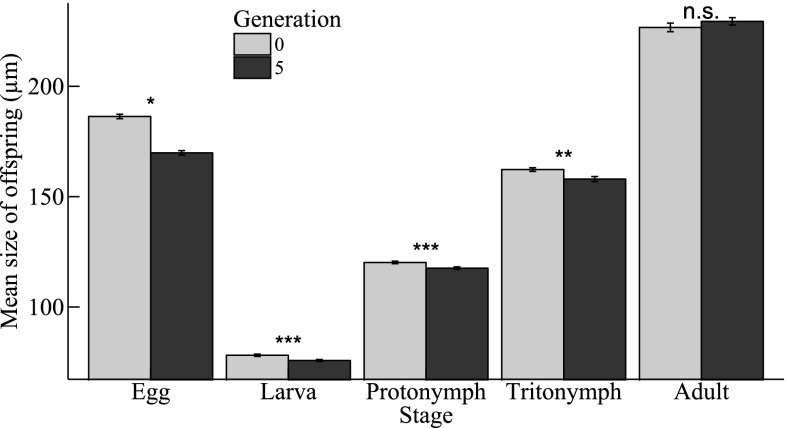
Mean (± SE) *Rhizoglyphus robini* life stage size (µm) of offspring from generations 0 and 5 (*: 0.01 < *p* < 0.05, **: 0.001 < *p* < 0.01, ***: *p* < 0.001; n.s.: *p* > 0.05)

We found a significant interaction between selection line and generation number on the mean length of eggs (G × L: χ^2^ = 4.221, *df* = 1, *P* = 0.040, *n* = 262): the mean length of eggs produced in fighter-selected lines was longer than those produced in scrambler-selected lines in generation 0. However, in generation 5 we found the opposite effect, as eggs produced in scrambler lines were longer than those produced in fighter lines (Fig. 3). Notably, the mean egg length of both selection lines decreased between generations 0 and 5 (Fig. 3). Besides eggs decreasing in length between the first and final generation, we found similar declines over time in the structural sizes of larvae, protonymphs and tritonymphs (Table 1; Fig. 4).

**Table 1:** Changes in the sizes of *Rhizoglyphus robini* larvae, protonymphs and tritonymphs with the number of generations of selection (G)

Stage	Estimate ($\hat {e}$)	SE	χ^2^ (*df* = 1)	*p*	*n*
Larva	− 2.440	0.703	11.877	< 0.001	261
Protonymph	− 2.839	0.845	10.969	< 0.001	246
Tritonymph	− 4.797	1.488	10.351	0.001	260

Parameter estimates show the difference in the intercept between generations 5 and 0 (G: Δ generation 5–0)

Furthermore, we found that larvae in scrambler lines were significantly smaller than larvae in fighter lines (L: Δ scramblers − fighters: $\hat {e}$ = −1.952, SE = 0.703; χ^2^ = 7.683, *df* = 1, *P* = 0.006, *n* = 261). Female mites (adult and juvenile) were larger than mites of the same life stage that were or would become fighters, which in turn were larger than mites of the same life stage that were or would become scramblers. Specifically, this difference was found in protonymphs, tritonymphs and adults (Table 2).

**Table 2:** Effects of the sex or morph of a *Rhizoglyphus robini* offspring individual (O) on the sizes of protonymphs, tritonymphs and adults

Stage	Δ	Estimate ($\hat {e}$)	SE	χ^2^ (*df* = 1)	*p*	*n*
Protonymph	Scramblers − females	− 4.553	1.235	16.018	< 0.001	246
	Fighters − females	− 2.498	0.949			
Tritonymph	Scramblers − females	− 9.532	2.184	21.840	< 0.001	260
	Fighters − females	− 5.084	1.639			
Adult	Scramblers − females	− 35.454	2.989	146.160	< 0.001	259
	Fighters − females	− 23.499	2.228			

Parameter estimates show the difference in the intercept of scramblers compared to females (Δ scramblers − females) or fighters compared to females (Δ fighters − females)

**Fig. 5:**
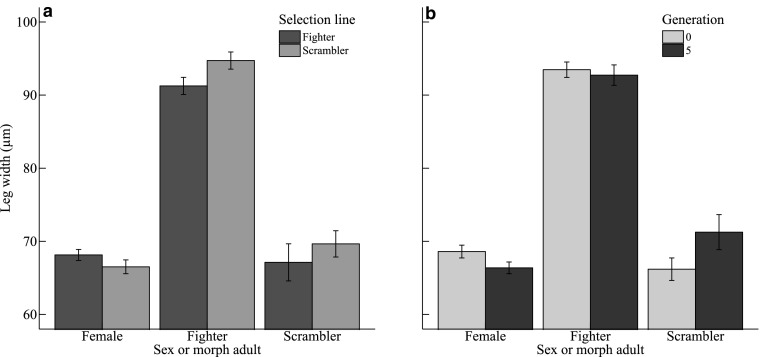
Mean (± SE) third leg pair width (µm) in *Rhizoglyphus robini* adult females, fighters and scramblers compared between **a** scrambler vs. fighter lines, and **b** generation 0 vs. 5

The width of the third leg pair of adults was affected by two two-way interactions. Firstly, there was a statistically significant interaction between the selection line and the sex or morph of the adult offspring on the width of the third leg pair (L × O: χ^2^ = 6.290, *df* = 2, *P* = 0.043, *n* = 259): the legs of fighters and scramblers from scrambler lines were wider than those from fighter lines, but the reverse was true for legs of females (Fig. 5a). Secondly, the third leg pair width of adults was significantly affected by the interaction between the generation number and the sex or morph of the adults (G × O: χ^2^ = 7.677, *df* = 2, *P* = 0.022, *n* = 259): between the start and the end of the experiment, the third leg pair scramblers became wider and the leg width of females became smaller; the leg width of fighters remained more or less unchanged (Fig. 5b).

We tested for differences in allometry between body size and leg width between the two selection lines for females, scramblers and fighters. For this analysis we fitted the natural logarithm (ln) of the body size and leg width using a SMATR (Warton et al. 2012). We found that there was no difference between the two selection lines in ln[body size] × ln[leg width] allometry in the elevation or slope of females, scramblers or fighters (see *Online Appendix* Table A4).

Finally, we found no evidence that the duration of the various stages was affected by the selection line, generation number or the sex or morph the mite will develop into, nor by any of the two- or three-way interactions between these main effects (see *Online Appendix* Table A3.C for results).

### Clutch size and composition

Clutch size, as well as the number of mites of most life stages within a clutch, decreased over the course of the selection experiment, for measurements taken on both day 5 and day 12. In the day-5 data, we found a negative correlation between the number of generations of selection and the clutch size (G: Δ generation 5 − 0: $\hat {e}$ = − 5.205, SE = 0.930; χ^2^ = 29.324, *df* = 1, *P* < 0.001, *n* = 234) and the number of eggs per clutch (G: Δ generation 5 − 0: $\hat {e}$ = −3.586, SE = 0.878; χ^2^ = 16.109, *df* = 1, *P* < 0.001, *n* = 234). Similarly, in the day 12 data, the clutch size decreased with the decreasing numbers of generations of selection (G: Δ generation 5 − 0: $\hat {e}$ = − 9.695, SE = 1.428; χ^2^ = 41.720, *df* = 1, *P* < 0.001, *n* = 223). Correspondingly, we found a decrease in the number of larvae, protonymphs, tritonymphs, females and fighters (Table 3).

**Table 3:** Changes in *Rhizoglyphus robini* clutch composition on day 12 as a result of the number of generations of selection (G)

Stage	Estimate ($\hat {e}$)	SE	χ^2^ (*df* = 1)	*p*	*n*
Larva	− 2.235	0.477	21.052	< 0.001	224
Protonymphs	− 3.358	0.623	27.246	< 0.001	224
Tritonymphs	− 2.747	0.503	28.056	< 0.001	223
Females	− 0.777	0.309	6.267	0.012	224
Fighters	− 0.662	0.213	9.595	0.002	224

Parameter estimates show the difference in the intercept of the number of larvae, protonymphs, tritonymphs, females and fighters per clutch in generations 0 vs. 5 (Δ generation 5 − 0)

**Fig. 6:**
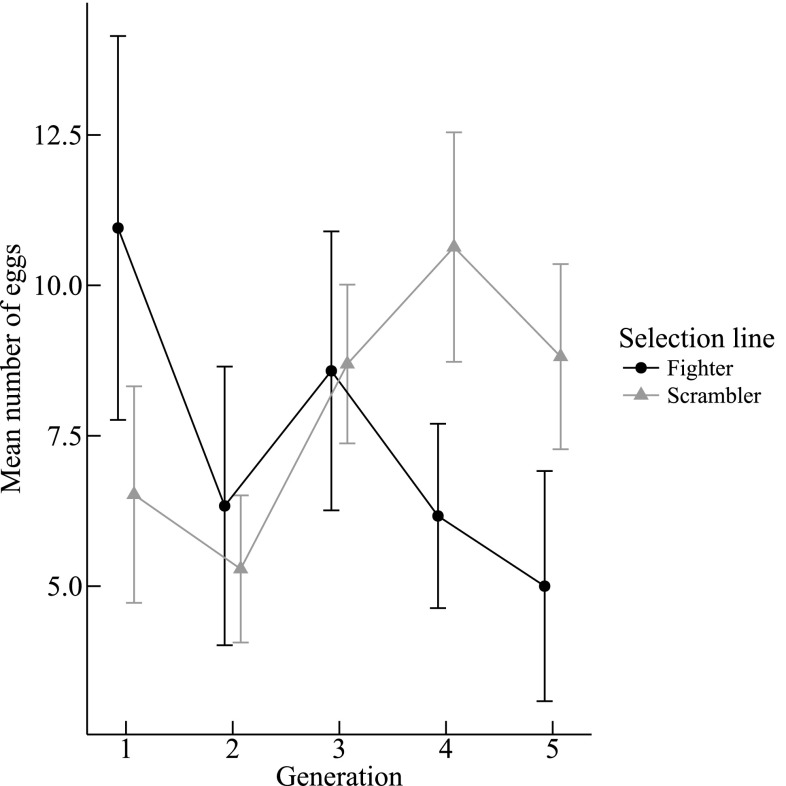
Mean (± SE) number of *Rhizoglyphus robini* eggs in each clutch counted on day 12 of each new generation

The number of larvae on day 5 were affected by a significant two-way interaction between the generation number and selection line (G × L: χ^2^ = 4.794, *df* = 1, *P* = 0.029, *n* = 234, *Online Appendix* Fig. A2). However, we are confident this effect was the result of the unusually large number of larvae produced by generation 1 of both selection lines, working as a leverage point (see *Online Appendix* Fig. A2). The number of eggs produced before day 12 was significantly affected by the interaction between the generation number and selection line (G × L: χ^2^ = 6.265, *df* = 1, *P* = 0.012, *n* = 223, Fig. 6). Figure 6 shows that the lines for egg production of both selection lines cross: the number of eggs is higher in fighter lines in the first two generations, but during generations 4 and 5, the number of eggs in scrambler lines becomes higher.

**Fig. 7:**
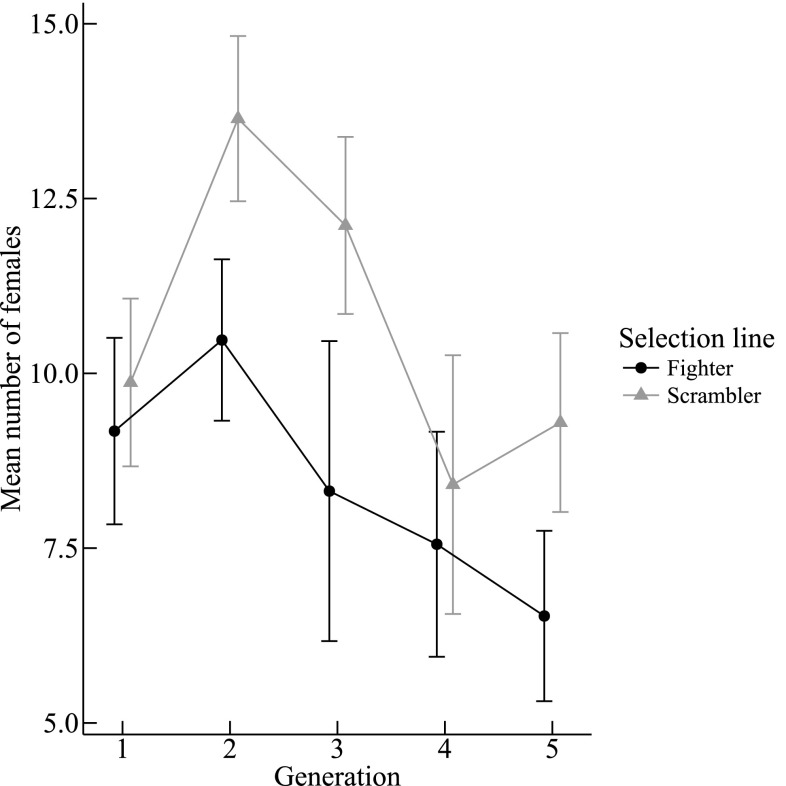
Mean (± SE) number of *Rhizoglyphus robini* females found on day 12 in each clutch over five generations

On day 12 we found more females in the clutches of scrambler lines than in fighter lines (L: Δ scramblers − fighters: $\hat {e}$ = 2.406, SE = 0.880; χ^2^ = 7.420, *df* = 1, *P* = 0.006, *n* = 224, Fig. 7). Moreover, there were fewer fighters in scrambler lines than in fighter lines (L: Δ scramblers − fighters: $\hat {e}$ = − 2.419, SE = 0.606; χ^2^ = 15.477, *df* = 1, *P* < 0.001, *n* = 224). The number of scramblers in a clutch was the result of an interaction between the generation number and selection line (G × L: χ^2^ = 4.116, *df* = 1, *P* = 0.042, *n* = 224, cf. Fig. 2) and was higher in scrambler lines than in fighter lines.

## Discussion

Intralocus sexual conflict can occur when a phenotypic trait that is expressed in both females and males has different fitness optima in the sexes but is governed by the same genes (Bonduriansky and Chenoweth 2009). In IASC, selection acts in each sex towards reaching its own trait optimum. The result is typically that in neither sex the optimal trait value is reached (Bonduriansky and Chenoweth 2009). In male-dimorphic species, each male morph has inherently different optimal trait values (Morris et al. 2013). These different trait values will generate a stronger or weaker IASC between males and females—depending on the mismatch with female optimal trait values. Previous results show that females from lines selected for high-fitness ‘major’ males often have a lower fecundity than daughters from low-fitness ‘minor’ male selection lines (Harano et al. 2010; Plesnar-Bielak et al. 2014). Here, we investigated how the selective environment—five generations of bidirectional selection for male morph—affected life-history traits in terms of clutch size and composition, maturation time, expression of fighter-limited weaponry in scramblers and females, and life stage sizes and duration.

In our experiment, selection for male morph was successful and several correlated life-history traits differed between the selection lines. We confirmed previous findings that male morph expression in the bulb mite is heritable (Radwan 2003a; Smallegange and Coulson 2011; Plesnar-Bielak et al. 2014). Unsurprisingly, since heritability measures are strongly context-dependent (Falconer and Mackay 1996; Radwan 2009), our heritability values in scrambler (0.298) and fighter lines (0.419) differed from those reported for other populations: Radwan (2003a): fighter lines 0.18–0.39; scrambler lines 0.79–0.83; Smallegange and Coulson (2011): fighter lines 0.30, scrambler lines 0.41.

In line with some previous studies (Harano et al. 2010; Plesnar-Bielak et al. 2014), we found some potential fitness benefits of females in lines selected against male weaponry. A correlated response to our selection regime that we found was that fighter-line females produced more eggs in the first generation, but scrambler-line females laid more eggs during the fourth and fifth generation. The fact that females maintained a higher egg-laying rate in scrambler lines could be the result of a stronger IASC between females and fighters, than between females and scramblers (Stuglik et al. 2014; Plesnar-Bielak et al. 2014). The latter result is consistent with the findings of Plesnar-Bielak et al. (2014) and Harano et al. (2010), that female fecundity was lower in lines selected for male weapons than in lines selected against male weapons. Hence, the putative lower IASC levels in scrambler lines may have beneficial effects on female fitness.

Some correlated responses to our selection regime may have important effects on how scrambler and fighter fitness vary under different environmental conditions. This is in addition to the fact that scramblers of the same cohort mature earlier than fighters (Smallegange 2011), which is beneficial in growing—as opposed to declining—populations (Caswell 1982). Higher egg-laying rates of scrambler-related females can also be advantageous in growing populations when competition for resources is limited (Wilbur et al. 1974). Also—to our knowledge for the first time in a bidirectional selection-line experiment—we reported that scrambler-line clutches consistently produced more females than clutches of fighter lines. Possibly, this was a demographic response if juvenile or adult females were killed more frequently by the larger number of fighters in fighter lines (cf. Van den Beuken and Smallegange 2018). The higher number of females likely yields a high clutch growth rate in growing populations (Lee et al. 2011). All this means that we expect scramblers to perform better in growing populations. In turn, we expect fighters to perform better in small populations where fighters use their weapons to monopolize access to females by killing rivals (Radwan and Klimas 2001). Because bulb mite populations likely show boom-bust cycles, given the ephemeral nature of their food source (Lesna et al. 1996), the maintenance of male-dimorphisms could be fuelled by frequent boom-bust cycles where fighter and scrambler relative fitness advantages fluctuate, in addition to their differences in success in intrasexual competition (Radwan 2009).

Generally, the evolutionary shift in male morph expression that we observed in our artificial selection experiment did not carry over to offspring life-history traits. We did find a significant interaction between selection line and generation number on egg length: eggs from fighters were initially longer than those from scramblers (as in Smallegange 2011). However, after five generations of selection, eggs produced in scrambler lines were longer. In contrast, larvae from fighter lines were on average larger than larvae from scrambler lines, regardless of the generation number. In subsequent life stages, we found no differences in the structural body sizes between the selection lines. Selection for scrambler or fighter expression therefore had no carry-over effects on adult body size. This is in line with the environmental threshold model (Hazel et al. 1990, 2004) that assumes that (adult) body size distributions remain unaltered as male morph expression evolves. Instead, the model predicts that, within a constant body size distribution, the threshold for male morph expression shifts in response to a selection pressure (e.g., Tomkins et al. 2011), resulting in more or fewer males developing into a fighter. Such an evolutionary shift in the threshold for male morph expression likely occurred in our selection experiment (cf. Smallegange and Deere 2014), with limited carry-over effects on juvenile sizes.

We observed that the mean sizes of life stages and the number of individuals present per life stage, as well as the mean clutch size, decreased over the course of the selection experiment regardless of the selection line. Interestingly, although the mean size of all juvenile stages decreased over the course of the selection experiment, the mean size of adults did not decrease. Perhaps mites in the final juvenile stage were able to compensate for any retarded growth (Hector and Nakagawa 2012). Alternatively, smaller individuals may have died before reaching adulthood, biasing the body size data (the *number* of adult females and fighters also decreased with increasing number of generations).

The general decrease in clutch sizes (and some individual life stage sizes) may be the adverse result of inbreeding or genetic drift which was, to some extent, unavoidable in our experimental setup (the lowest inbreeding coefficient in our replicates was 0.137, the highest was 0.309). Inbreeding and genetic drift can have had several unknown effects on our results, possibly even interacting with traits selected for in selection lines. High levels of inbreeding in *R. robini* are known to reduce female fecundity, increase juvenile mortality and, consequently, increase the risk of population extinction (Radwan 2003b). However, we contest that the selection-line-specific differences in life-history traits that we observed are the result of inbreeding, and not of bidirectional selection. We likely selected for the threshold (or sensitivity to environmental cues) for male morph expression to increase or decrease, respectively, in scrambler and fighter lines. If this selection results in higher levels of inbreeding (i.e., lower genetic variation) in for instance fighter lines, then this would entail that there is less standing genetic variation in genes that would decrease the threshold (resulting in more fighters) than there is in genes that would increase the threshold. To our knowledge, there are no empirical studies to support this idea, and we think that it is unlikely that it occurred in our study.

We also inspected the allometric relation between body size and leg width to test if the scaling between body size and leg width differed between selection lines. Buzatto et al. (2018) found that bidirectional selection for leg width in *R. echinopus* fighters resulted in positively correlated responses in leg sizes of scramblers and females. This suggests that the development of leg size is not fully decoupled between fighters and scramblers (also see Pike et al. 2017) or females. Though we did not select for leg width in this experiment, we did find that in scrambler lines, compared to fighter lines, fighter and scrambler legs were wider and female legs were narrower. However, contrary to our hypothesis, we could not support the notion that this was due to differences in allometric scaling between legs and body size. Regardless, given our results and those of Buzatto et al. (2018), we stress that the expression of traits that are considered to be limited to one sex or male morph may be expressed (to a limited extent) in other sexes or morphs. These traits should therefore not be ignored as such male-specific traits may affect fecundity in females (e.g., Swierk and Langkilde 2013).

In conclusion, our data show that multiple generations of bidirectional selection for male morph can result in correlative responses in other life-history traits, including shifts in the number of females within clutches, and the long-term egg-laying rate and egg sizes. Differences between selection lines in these life-history traits may define under what circumstances fighter and scrambler clutches may have an increased fitness. Likely, the differences between selection lines were fuelled by different levels of IASC between females and the two male morphs.

## Electronic supplementary material

Below is the link to the electronic supplementary material.

Supplementary material 1 (PDF 448 KB)
