# Supplementary material for: Life-history consequences of bidirectional selection for male morph in a male-dimorphic bulb mite
Source: Exp Appl Acarol. 2018 Nov 12;76(4):435–52. doi: 10.1007/s10493-018-0320-5 (PMC6280856; doi:10.1007/s10493-018-0320-5)
Supplement: Supplementary file 1 — Supplementary material 1 (PDF 448 KB) [file 10493_2018_320_MOESM1_ESM.pdf]

Online Appendix

Supporting Information to:

Life-history consequences of bidirectional selection for male morph in a male-dimorphic bulb mite

In: Experimental and Applied Acarology

Tom P.G. Van den Beuken (TvdB)[1]* and Isabel M. Smallegange (IMS)[1]

[1] Department of Evolutionary and Population Biology, Institute for Biodiversity and Ecosystem Dynamics, University of Amsterdam, PO Box 94240, 1090 GE Amsterdam, the Netherlands

*Corresponding author:
Tom P.G. van den Beuken
E-mail address: t.p.g.vandenbeuken@outlook.com

We conducted an experiment in which we bidirectionally selected for male morph expression for five generations in the male-dimorphic bulb mite *Rhizoglyphus robini*. In generations 1 to 5 we counted how many males of each morph were present in each clutch on day 12 (after the first adults emerged). With these data we calculated the heritability of the liability of the scrambler and fighter morph (Table A2). Additionally, we tested how the proportion fighters of males was affected by the selection line, the number of generations and their two-way interaction (Table A3.A).

Using data obtained by following three eggs from each clutch in generations 0 and 5 to adulthood (replicate numbers in Table A1.A), we tested for the effects of selection line,

generation number and (eventual) morph or sex of mites, as well as the three two-way interactions and single three-way interaction, on the i) life stage size (Table A3.B), ii) leg width of each adult (Table A3.B) and the iii) life stage duration and maturation time (Table A3.C). For an example of the structural size and leg width measurement, see Fig. A2.

We assessed the clutch size and composition twice per generation (on day 5 and day 12, replicate numbers in Table A1.B). We used this data to analyse how the selection line, generation number and their two-way interaction affect the clutch size and composition as assessed on day 5 (Table A3.D) and day 12 (Table A3.E). Fig. A2 shows the outliers we found in the number of larvae found in clutches on day 5, in both selection lines.

For all models we used model simplification steps to find a minimal adequate model; the performed simplification steps can be found in Table A3.

Tables

Table A1. The number of replicates for the clutch size and composition assay (A) and the life stage and duration, third leg size and developmental time assay (B). Table A shows the number of isolated offspring that could be tracked from egg to adult in generations 0 and 5. Table B shows the number of clutches that were assessed per selection line and generation (generations 1 to 5). These assessments were made twice in each generation: on day 5 and day 12.

A	**Fighter line**		**Scrambler line**	
Generation	0	5	0	5
Replicates	64	63	66	68

B	**Fighter line**					**Scrambler line**				
Generation	1	2	3	4	5	1	2	3	4	5
Day 5	24	22	21	19	22	23	28	25	23	27
Day 12	22	21	19	18	17	23	28	26	22	27

Table A2. Calculation of the narrow-sense heritability of the liability of the scrambler and fighter morph in respectively scrambler- and fighter lines. The presented fractions (*f*) are the fraction of males that developed the morph selected for as counted on day 12 of each generation. The mean liability for a trait (*x*) and the mean deviation of individuals from the mean liability (*i*) are both expressed in standard-deviation units from the population mean (Falconer and Mackay, 1996). The values for *x* and *i* were both obtained from Falconer and Mackay (1996). The heritability of liability were calculated using the formulae in Falconer and Mackay (1996):
$\boldsymbol{Heritability} = \boldsymbol{R}/\sum \boldsymbol{i}$; with R being the response to selection between generation 1 and generation 5: $\boldsymbol{R} = -(\boldsymbol{x_5} - \boldsymbol{x_1})$. In several populations no males were present on day 12 (in each generation and selection line: 30 – *n*); these populations were omitted for this calculation.

	**Scrambler line**				**Fighter line**			
**Generation**	***n***	***f***	***x***	***i***	***n***	***f***	***x***	***i***
1	20	0.349	0.385	1.058	20	0.900	−1.282	0.195
2	26	0.525	−0.050	0.751	21	0.883	−1.175	0.221
3	23	0.512	−0.025	0.777	12	0.902	−1.282	0.191
4	17	0.673	−0.440	0.533	15	0.896	−1.282	0.204
5	24	0.747	−0.674	0.431	14	0.953	−1.665	0.103
	R = 1.059, Heritability = 0.145				R = 0.383, Heritability = 0.538			

Table A3. Model simplification steps used to analyse (A) the relative number of fighters in clutches (B) the size of each life stage and the width of the third leg pair of adults, (C) the life stage duration and total development time, (D) the clutch size and composition on day 5 and (E) day 12. From each fitted model (model 1), we removed the least significant term of the highest order interaction (term underscored) to produce a reduced model (model 2). To test for a significant difference in deviance between the two models, we used a likelihood ratio test compared to a chi-squared distribution. If the reduced model had a significantly higher deviance χ^2 than the fitted model ($P < 0.05$, printed in bold), then we kept the removed term in the next fitted model and removed the second least significant term from the model to produce a new reduced model. We continued repeating these steps until only terms remained in the model of which the removal leads to a significant increase in deviance. The relative male morph number (Table A3.A) and clutch size and composition (Table A3.D and A3.E) analyses feature a two-way interaction between the generation number (G) and selection line (L) (G × L). For the analyses of the mean sizes per stage (A3.B) and stage duration (A3.C) we used a three-way interaction between the generation number, selection line and the sex or morph into which a mite develops (O) (G × L × O). The two-way interactions (G × L, G × O and L × O) and the three main effects (G, L and O) are included in each model where the three-way interaction G · L · O is used. Similarly, the two main effects in the two-way interaction G · L are also included in each model. Each model includes the replicate block number as a random factor; this random factor was never removed from the model. We used linear mixed-effects models (LMM) for all analyses except for the male morph ratio and the life stage duration and maturation time analysis (Table A3.C). The analyses of stage duration and male morph ratio did not produce normally distributed data; hence we used a generalized linear mixed-effects model (GLMM) with a Poisson error structure. We will show the parameter estimates ($\hat{e}$) of each significant main effect. The parameter estimates of selection line (L) are the intercepts of the scrambler lines (L-Scramblers) relative to those of the fighter lines ('L: Δ scramblers – fighters' in the main text). Likewise, parameter estimates of the main effect 'offspring morph or sex' (O) show the intercepts of mites that will become or are scramblers (O-Scramblers) or fighters (O-Fighters), relative to the intercepts of females (respectively 'O: Δ scramblers – females' and 'O: Δ fighters – females' in the main text). The parameter estimates of the main effect 'Generation' show the difference in intercept between generation 5 and generation 0 ('G: Δ generation 5 – 0' in the main text).

(A) Male morph ratio

Number of fighters, relative to total number of males after 12 days

Full model: GLMM: Number of fighters ~ G · L + offset (number of males). *N* = 271

**Step #**	**Model 1**	**Model 2**	χ^2	***P***
1	G · L	G + L	284.140	**< 0.001**
AIC-value (df)	15012.290 (5)	15294.430 (4)		

Final model: Number of fighters ~ G · L

(B) Life stage sizes

Mean length of eggs

Full model: LMM: Egg length ~ G · L · O. *N* = 262

**Step #**	**Model 1**	**Model 2**	χ^2	***P***
1	G · L · O	G · L + G · O + L · O	2.292	0.318
AIC-value (df)	1996.027 (14)	2005.847 (12)		
2	G · L + G · O + L · O	G · L + G · O	0.176	0.916
AIC-value (df)	2005.847 (12)	2010.857 (10)		
3	G · L + G · O	G · L + O	2.283	0.319
AIC-value (df)	2010.857 (10)	2017.843 (8)		
4	G · L + O	G + L + O	4.221	**0.040**
AIC-value (df)	2017.843 (8)	2023.917 (7)		
5	G · L + O	G · L	1.880	0.391
AIC-value (df)	2017.843 (8)	2021.675 (6)		

Final model: Egg length ~ G · L

Mean structural size of larvae

Full model: LMM: Size larvae ~ G · L ·O. $N = 261$

**Step #**	**Model 1**	**Model 2**	χ^2	$\boldsymbol{P}$
1	G · L · O	G · L + G · O + L · O	2.098	0.350
AIC-value (df)	1640.040 (14)	1646.960 (12)		
2	G · L + G · O + L · O	G · O + L · O	0.033	0.857
AIC-value (df)	1646.960 (12)	1647.548 (11)		
3	G · O + L · O	G · O + L	0.825	0.662
AIC-value (df)	1647.548 (11)	1650.335 (9)		
4	G · O + L	L + O + G	2.391	0.303
AIC-value (df)	1650.335 (9)	1654.700 (7)		
5	L + O + G	L + G	1.417	0.492
AIC-value (df)	1654.700 (7)	1655.330 (5)		
6	L + G	G	7.683	**0.006**
AIC-value (df)	1655.330 (5)	1662.091 (4)	$\hat{e} = -1.952$, SE = 0.703, $t = -2.780$	
7	L + G	L	11.877	**< 0.001**
AIC-value (df)	1655.330 (5)	1666.291 (4)	$\hat{e} = -2.440$, SE = 0.703, $t = -3.470$	

Final model: Size larvae ~ L + G

Mean structural size of protonymphs

Full model: LMM: Size protonymphs ~ G · L · O. N = 246

**Step #**	**Model 1**	**Model 2**	χ^2	***P***
1	G · L · O	G · L + G · O + L · O	0.196	0.907
AIC-value (df)	1614.164 (14)	1619.867 (12)		
2	G · L + G · O + L · O	G · L + L · O	0.280	0.869
AIC-value (df)	1619.867 (12)	1622.849 (10)		
3	G · L + L · O	G · L + O	2.781	0.249
AIC-value (df)	1622.849 (10)	1628.130 (8)		
4	G · L + O	G + L + O	2.669	0.102
AIC-value (df)	1628.130 (8)	1631.668 (7)		
5	G + L + O	G + O	< 0.001	0.986
AIC-value (df)	1631.668 (7)	1631.180 (6)		
6	G + O	G	16.018	**< 0.001**
AIC-value (df)	1631.180 (6)	1646.984 (4)	F: $\hat{e}$ = −2.498, SE = 0.949, t = −2.630<br>S: $\hat{e}$ = −4.553, SE = 1.235, t = −3.690	
7	G + O	O	10.969	**< 0.001**
AIC-value (df)	1631.180 (6)	1641.706 (5)	$\hat{e}$ = −2.839, SE = 0.845, t = −3.360	

Final model: Size protonymphs ~ G + O

Mean structural size of tritonymphs

Full model: LMM: Size tritonymphs ~ G · L · O. *N* = 260

**Step #**	**Model 1**	**Model 2**	χ^2	***P***
1	G · L · O	G · L + G · O + L · O	0.333	0.847
AIC-value (df)	2003.883 (14)	2012.013 (12)		
2	G · L + G · O + L · O	G · O + L · O	0.020	0.889
AIC-value (df)	2012.013 (12)	2014.088 (11)		
3	G · O + L · O	L · O + G	0.187	0.911
AIC-value (df)	2014.088 (11)	2019.208 (9)		
4	L · O + G	L + O + G	0.703	0.704
AIC-value (df)	2019.208 (9)	2024.876 (7)		
5	L + O + G	O + G	0.525	0.469
AIC-value (df)	2024.876 (7)	2026.034 (6)		
6	O + G	O	10.351	**0.001**
AIC-value (df)	2026.034 (6)	2036.897 (5)	$\hat{e} = -4.797$, SE = 1.488, $t = -3.220$	
7	O + G	G	21.840	< **0.001**
AIC-value (df)	2026.034 (6)	2049.824 (4)	F: $\hat{e} = -5.084$, SE = 1.639, $t = -3.100$<br>S: $\hat{e} = -9.532$, SE = 2.184, $t = -4.360$	

Final model: Size tritonymphs ~ G + O

Mean structural size of adults

Full model: LMM: Size adults ~ G · L · O. N = 259

**Step #**	**Model 1**	**Model 2**	χ^2	***P***
1	G · L · O	G · L + G · O + L · O	0.617	0.734
AIC-value (df)	2142.719 (14)	2152.362 (12)		
2	G · L + G · O + L · O	G · L + G · O	1.250	0.535
AIC-value (df)	2152.362 (12)	2159.784 (10)		
3	G · L + G · O	L + G · O	1.441	0.230
AIC-value (df)	2159.784 (10)	2163.792 (9)		
4	L + G · O	L + G + O	2.782	0.249
AIC-value (df)	2163.792 (9)	2172.738 (7)		
5	L + G + O	L + O	0.653	0.419
AIC-value (df)	2172.738 (7)	2174.604 (6)		
6	L + O	O	1.387	0.239
AIC-value (df)	2174.604 (6)	2177.254 (5)		
7	O	1	146.160	**< 0.001**
AIC-value (df)	2177.254 (5)	2326.633 (3)	F: $\hat{e}$ = −23.499, SE = 2.228, t = −10.550<br>S: $\hat{e}$ = −35.454, SE = 2.989, t = −11.860	

Final model: Size adults ~ O

Mean leg width of adults

Full model: LMM: Leg width adults ~ G · L · O. *N* = 259

**Step #**	**Model 1**	**Model 2**	χ^2	***P***
1	G · L · O	G · L + G · O + L · O	0.692	0.708
AIC-value (df)	1758.174 (14)	1764.744 (12)		
2	G · L + G · O + L · O	G · O + L · O	2.045	0.153
AIC-value (df)	1764.744 (12)	1767.839 (11)		
3	G · O + L · O	L · O + G	7.677	**0.022**
AIC-value (df)	1767.839 (11)	1778.445 (9)		
4	G · O + L · O	G · O + L	6.290	**0.043**
AIC-value (df)	1767.839 (11)	1776.925 (9)		

Final model: Leg width adults ~ G · O + L · O

(C) Life stage duration and maturation time

Egg stage duration

Full model: GLMM: Egg stage duration ~ G · L ·O (Poisson error distribution). $N = 262$

Step #	Model 1	Model 2	χ^2	***P***
1	G · L · O	G · L + G · O + L · O	0.454	0.797
AIC-value (df)	890.603 (13)	887.057 (11)		
2	G · L + G · O + L · O	G · L + L · O	0.086	0.958
AIC-value (df)	887.057 (11)	883.142 (9)		
3	G · L + L · O	L · O + G	0.197	0.658
AIC-value (df)	883.142 (9)	881.339 (8)		
4	L · O + G	L + O + G	0.282	0.869
AIC-value (df)	881.339 (8)	877.621 (6)		
5	L + O + G	O + G	0.007	0.935
AIC-value (df)	877.621 (6)	875.627 (5)		
6	O + G	G	0.206	0.902
AIC-value (df)	875.627 (5)	871.834 (3)		
7	G	1	1.628	0.202
AIC-value (df)	871.834 (3)	871.461 (2)		

Final model: egg stage duration ~ 1

Larva stage duration

Full model: GLMM: larval stage duration ~ G · L · O (Poisson error distribution). *N* = 251

**Step #**	**Model 1**	**Model 2**	χ^2	***P***
1	G · L · O	G · L + G · O + L · O	0.477	0.788
AIC-value (df)	757.953 (13)	754.430 (11)		
2	G · L + G · O + L · O	G · O + L · O	0.012	0.912
AIC-value (df)	754.430 (11)	752.4426 (10)		
3	G · O + L · O	L · O + G	0.416	0.812
AIC-value (df)	752.4426 (10)	748.859 (8)		
4	L · O + G	L + O + G	0.879	0.644
AIC-value (df)	748.859 (8)	745.738 (6)		
5	L + O + G	O + G	0.003	0.958
AIC-value (df)	745.738 (6)	743.740 (5)		
6	O + G	O	0.043	0.835
AIC-value (df)	743.740 (5)	741.784 (4)		
7	O	1	0.553	0.759
AIC-value (df)	741.784 (4)	738.336 (2)		

Final model: larval stage duration ~ 1

Protonymph stage duration

Full model: GLMM: protonymph stage duration ~ G · L · O (Poisson error distribution). *N* = 251

**Step #**	**Model 1**	**Model 2**	χ^2	***P***
1	G · L · O	G · L + G · O + L · O	0.087	0.958
AIC-value (df)	675.502 (13)	671.589 (11)		
2	G · L + G · O + L · O	G · L + G · O	0.131	0.937
AIC-value (df)	671.589 (11)	667.7201 (9)		
3	G · L + G · O	G · L + O	0.210	0.900
AIC-value (df)	667.7201 (9)	663.930 (7)		
4	G · L + O	G + L + O	0.090	0.764
AIC-value (df)	663.930 (7)	662.020 (6)		
5	G + L + O	G + L	0.036	0.982
AIC-value (df)	662.020 (6)	658.056 (4)		
6	G + L	L	0.100	0.752
AIC-value (df)	658.056 (4)	656.156 (3)		
7	L	1	0.154	0.695
AIC-value (df)	656.156 (3)	654.310 (2)		

Final model: protonymph stage duration ~ 1

Tritonymph stage duration

Full model: GLMM: tritonymph stage duration ~ G · L · O (Poisson error distribution). $N = 261$

**Step #**	**Model 1**	**Model 2**	χ^2	***P***
1	G · L · O	G · L + G · O + L · O	0.239	0.887
AIC-value (df)	767.252 (13)	763.491 (11)		
2	G · L + G · O + L · O	G · L + L · O	0.017	0.992
AIC-value (df)	763.491 (11)	759.508 (9)		
3	G · L + L · O	G · L + O	0.291	0.865
AIC-value (df)	759.508 (9)	755.799 (7)		
4	G · L + O	G + L + O	0.312	0.577
AIC-value (df)	755.799 (7)	754.111 (6)		
5	G + L + O	G + L	0.153	0.926
AIC-value (df)	754.111 (6)	750.264 (4)		
6	G + L	G	0.095	0.757
AIC-value (df)	750.264 (4)	748.359 (3)		
7	G	1	1.355	0.244
AIC-value (df)	748.359 (3)	747.714 (2)		

Final model: tritonymph stage duration ~ 1

Maturation time

Full model: GLMM: maturation time ~ G · L ·O (Poisson error distribution). *N* = 261

**Step #**	**Model 1**	**Model 2**	χ^2	***P***
1	<u>G · L · O</u>	G · L + G · O + L · O	0.045	0.978
AIC-value (df)	1131.123 (13)	1127.169 (11)		
2	G · L + <u>G · O</u> + L · O	G · L + L · O	0.096	0.953
AIC-value (df)	1127.169 (11)	1123.265 (9)		
3	G · L + <u>L · O</u>	G · L + O	0.244	0.885
AIC-value (df)	1123.265 (9)	1119.508 (7)		
4	<u>G · L</u> + O	G + L + O	0.236	0.627
AIC-value (df)	1119.508 (7)	1117.744 (6)		
5	G + L + <u>O</u>	G + L	0.138	0.933
AIC-value (df)	1117.744 (6)	1113.883 (4)		
6	G + <u>L</u>	G	0.115	0.735
AIC-value (df)	1113.883 (4)	1111.998 (3)		
7	<u>G</u>	1	1.134	0.287
AIC-value (df)	1111.998 (3)	1111.131 (2)		

Final model: maturation time ~ 1

(D) Clutch size and composition – day 5

Clutch size – day 5

Full model: LMM: Clutch size ~ G · L. *N* = 234

**Step #**	**Model 1**	**Model 2**	χ^2	***P***
1	<u>G · L</u>	G + L	0.375	0.375
AIC-value (df)	2076.157 (6)	2078.041 (5)		
2	G + <u>L</u>	G	0.799	0.372
AIC-value (df)	2078.041 (5)	2080.625 (4)		
3	<u>G</u>	1	29.324	< **0.001**
AIC-value (df)	2080.625 (4)	2109.720 (3)	$\hat{e}$ = −5.205, SE = 0.930, *t* = −5.594	

Final model: Clutch size ~ G

Number of eggs – day 5

Full model: LMM: Number of eggs ~ G · L. *N* = 234

**Step #**	**Model 1**	**Model 2**	χ^2	***P***
1	<u>G · L</u>	G + L	0.227	0.634
AIC-value (df)	2050.671 (6)	2051.878 (5)		
2	G + <u>L</u>	G	0.228	0.633
AIC-value (df)	2051.878 (5)	2053.782 (4)		
3	<u>G</u>	1	16.109	< **0.001**
AIC-value (df)	2053.782 (4)	2069.517 (3)	$\hat{e}$ = −3.586, SE = 0.878, *t* = −4.085	

Final model: Number of eggs ~ G

Number of larvae – day 5

Full model: LMM: Number of larvae ~ G · L. N = 234

Step #	Model 1	Model 2	χ^2	***P***
1	G · L	G + L	4.794	**0.029**
AIC-value (df)	1327.914 (6)	1330.534 (5)		

Final model: Number of larvae ~ G · L

(E) Clutch size and composition – day 12

Clutch size – day 12

Full model: LMM: Clutch size ~ G · L. N = 223

Step #	Model 1	Model 2	χ^2	***P***
1	G · L	G + L	0.287	0.592
AIC-value (df)	2153.526 (6)	2155.780 (5)		
2	G + L	G	0.2422	0.623
AIC-value (df)	2155.780 (5)	2158.666 (4)		
3	G	1	41.720	**< 0.001**
AIC-value (df)	2158.666 (4)	2201.071 (3)	$\hat{e}$ = −9.695, SE = 1.428, t = −6.789	

Final model: Clutch size ~ G

Number of eggs – day 12

Full model: LMM: Number of eggs ~ G · L. *N* = 223

**Step #**	**Model 1**	**Model 2**	χ^2	***P***
1	G · L	G + L	6.265	**0.012**
AIC-value (df)	1618.361 (6)	1624.052 (5)		

Final model: Number of eggs ~ G · L

Number of larvae – day 12

Full model: LMM: Number of larvae ~ G · L. *N* = 224

**Step #**	**Model 1**	**Model 2**	χ^2	***P***
1	G · L	G + L	0.002	0.970
AIC-value (df)	1675.405 (6)	1675.169 (5)		
2	G + L	G	0.825	0.364
AIC-value (df)	1675.169 (5)	1676.437 (4)		
3	G	1	21.052	**< 0.001**
AIC-value (df)	1676.437 (4)	1695.786 (3)	$\hat{e}$ = −2.235, SE = 0.477, *t* = −4.688	

Final model: Number of larvae ~ G

Number of protonymphs – day 12

Full model: LMM: Number of protonymphs ~ G · L. $N = 224$

**Step #**	**Model 1**	**Model 2**	χ^2	***P***
1	G · L	G + L	1.746	0.186
AIC-value (df)	1796.525 (6)	1798.515 (5)		
2	G + L	G	3.538	0.060
AIC-value (df)	1798.515 (5)	1803.001 (4)		
3	G	1	27.246	< **0.001**
AIC-value (df)	1803.001 (4)	1829.215 (3)	$\hat{e} = -3.358$, SE = 0.623, $t = -5.387$	

Final model: Number of protonymphs ~ G

Number of tritonymphs – day 12

Full model: LMM: Number of tritonymphs ~ G · L. $N = 224$

**Step #**	**Model 1**	**Model 2**	χ^2	***P***
1	G · L	G + L	0.250	0.617
AIC-value (df)	1706.958 (6)	1707.081 (5)		
2	G + L	G	0.589	0.443
AIC-value (df)	1707.081 (5)	1708.225 (4)		
3	G	1	28.056	< **0.001**
AIC-value (df)	1708.225 (4)	1734.787 (3)	$\hat{e} = -2.747$, SE = 0.503, $t = -5.466$	

Final model: Number of tritonymphs ~ G

Number of females – day 12

Full model: LMM: Number of females ~ G · L. $N = 224$

**Step #**	**Model 1**	**Model 2**	χ^2	$\boldsymbol{P}$
1	G · L	G + L	0.001	0.980
AIC-value (df)	1488.917 (6)	1487.806 (5)		
2	G + L	L	6.267	**0.012**
AIC-value (df)	1487.806 (5)	1491.567 (4)	$\hat{e} = -0.777$, SE = 0.309, $t = -2.519$	
3	G + L	G	7.420	**0.006**
AIC-value (df)	1487.806 (5)	1494.778 (4)	$\hat{e} = 2.406$, SE = 0.880, $t = 2.736$	

Final model: Number of females ~ G + L

Number of fighters – day 12

Full model: LMM: Number of fighters ~ G · L. $N = 224$

**Step #**	**Model 1**	**Model 2**	χ^2	$\boldsymbol{P}$
1	G · L	G + L	2.481	0.115
AIC-value (df)	1319.004 (6)	1319.641 (5)		
2	G + L	L	9.595	**0.002**
AIC-value (df)	1319.641 (5)	1325.909 (4)	$\hat{e} = -0.662$, SE = 0.213, $t = -3.114$	
3	G + L	G	15.477	< **0.001**
AIC-value (df)	1319.641 (5)	1333.921 (4)	$\hat{e} = -2.419$, SE = 0.606, $t = -3.994$	

Final model: Number of fighters ~ G + L

Number of scramblers – day 12

Full model: LMM: Number of scramblers ~ G · L. $N = 224$

**Step #**	**Model 1**	**Model 2**	χ^2	***P***
1	<u>G · L</u>	G + L	4.116	**0.042**
AIC-value (df)	1185.395 (6)	1186.996 (5)		

Final model: Number of scramblers ~ G · L

Table A4. Results of the SMATR analysis testing for differences in allometry between body size and leg width between females, scramblers and fighters in both selection lines. Shown are the elevation and slope (with the 95% confidence interval [CI]) of the ln body size × ln leg width allometry of females, scramblers and fighters in fighter and scrambler lines.

Sex or morph	Selection line	Elevation (95% CI)	Slope (95% CI)
Female	Fighter	4.304 (3.278, 5.331)	0.282 (0.041, 0.558)
	Scrambler	4.265 (3.429, 5.101)	0.289 (0.093, 0.508)
Scrambler	Fighter	2.490 (1.056, 3.923)	0.673 (0.363, 1.109)
	Scrambler	2.151 (0.579, 3.723)	0.750 (0.418, 1.237)
Fighter	Fighter	0.936 (−2.386, 4.258)	0.987 (0.374, 2.572)
	Scrambler	2.654 (1.122, 4.186)	0.600 (0.289, 1.028)

Figures

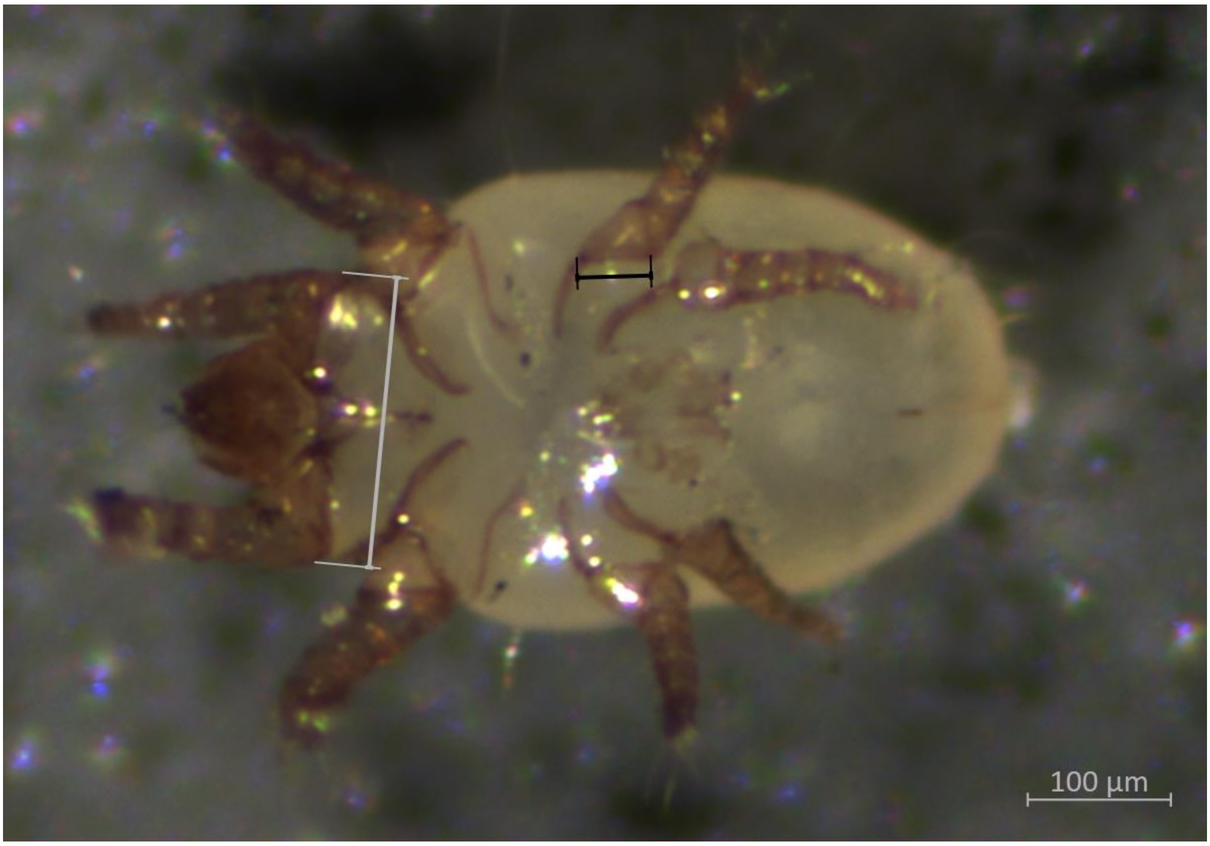

Fig. A1 Structural body size (grey) and third leg width (black) measurements on the ventral side of a left-facing female

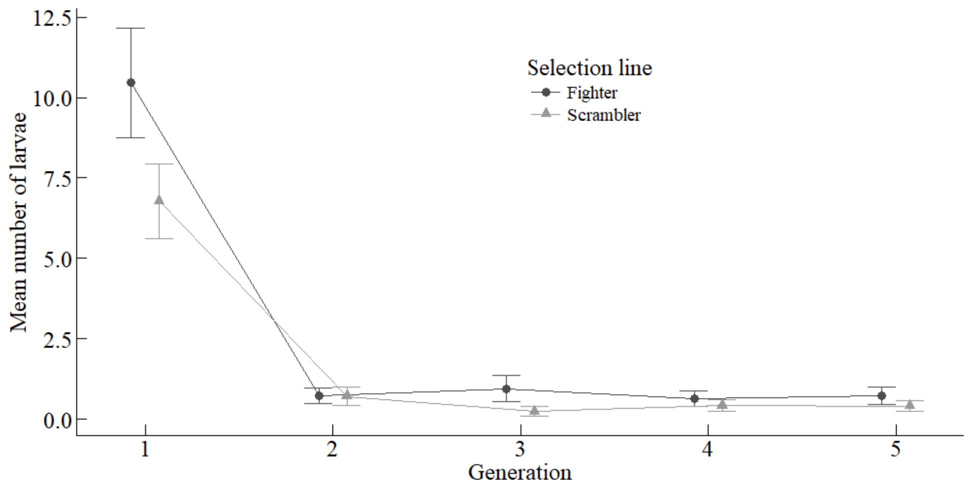

Fig. A2 The number of larvae ± SE as counted on day 5 for generations 1 to 5
